# Choice of randomisation time-point in non-inferiority studies of reduced treatment duration: experience from the SCOT study

**DOI:** 10.1186/1745-6215-12-S1-A30

**Published:** 2011-12-13

**Authors:** Jim Paul, Tim Iveson, Rachel Midgely, Andrea Harkin, Michelle Masterton, Laura Alexander, Jim Cassidy

**Affiliations:** 1 Cancer Research UK Clinical Trials Unit Glasgow, Glasgow, G12 0YT, UK; 2 Southampton University Hospitals NHS Trust, Southampton, SO16 6YD, UK; 3 Department of Clinical Pharmacology, Oxford, OX3 7DQ, UK; 4 Oxford Clinical Trials Office, Oxford, OX3 7DQ, UK

## Background

In a non-inferiority study where the aim is to compare a reduced treatment duration there is a choice of two randomisation time-points. Patients can be randomised at the end of the reduced treatment period to continue or not to the standard duration. This is optimum for maximising compliance, but has the disadvantage for the patient that treatment duration is not known from the outset. It is also more difficult to implement and, because of patients dropping out prior to randomisation, may result in an unrepresentative patient group. The alternative is to randomise patients prior to starting treatment.

## Methods

SCOT is a large (9500) phase III randomised non-inferiority study comparing 6 versus 3 months adjuvant treatment in colorectal cancer. In the first year of recruitment centres were randomised to either randomise patients prior to treatment (Up-front:U) or after they had completed 3 months of treatment (Delayed:D). In June 2009 the performance of the two approaches was reviewed in terms of recruitment rate, compliance and drop-out by the Trial Steering Committee (TSC) and the recommendation was made to change all sites to U. Using data to the end of 2010 we have looked at recruitment before/after the change, restricted to centres open >3 months prior to the change date. Updated drop-out information is also provided.

## Results

215 patients were registered for D and of these 159 were randomised; a drop-out rate of 26% (95% confidence interval [ci] 21-32%). This drop-out rate is higher than the general rate of patients stopping treatment at 3 months 18% (95% ci 16-21%).

For the 41 centres allocated to D the median [interquartile-range] randomisation rate prior to changing to U was 4.09 [1.29-7.09] patients/centre/year; after changing to U the equivalent figures were 8.87 [4.77 – 15.34]. This increase is statistically significant (p<.001).

For the 36 centres allocated to U the median [interquartile-range] randomisation rate prior to the date the D centres changed to U was 5.21 [3.56-11.55] patients/centre/year; post this date the equivalent figures were 7.52 [2.22 – 14.31]. This increase was not statistically significant (p=.121).

The increase in recruitment rate in the D centres is much higher than in the U centres post the randomisation change date (p=.001, test for interaction).

## Conclusions

In 2009 the TSC recommended that SCOT continue with up-front randomisation only. This was based on the higher randomisation rate with U and a high drop-out rate with D. Further data has endorsed that recommendation.

The SCOT study is supported by a trial grant from the MRC.

Cancer Research UK supports the work of the Glasgow Clinical Trials via a programme grant.

